# Esomeprazole Potentiates the Cytotoxic Effects of Cisplatin in Gastric Carcinoma Cells

**DOI:** 10.1002/jbt.70441

**Published:** 2025-08-11

**Authors:** Ziad Joha, Oğuzhan Kalkan, Fatih Yulak, Mustafa Ergül, Mustafa Asım Gedikli

**Affiliations:** ^1^ Department of Pharmacology, Faculty of Pharmacy Sivas Cumhuriyet University Sivas Turkey; ^2^ Department of Emergency Pursaklar State Hospital Ankara Turkey; ^3^ Department of Physiology, School of Medicine Sivas Cumhuriyet University Sivas Turkey; ^4^ Department of Biochemistry Sivas Cumhuriyet University School of Pharmacy Sivas Turkey; ^5^ Department of Internal Medicine, School of Medicine Sivas Cumhuriyet University Sivas Turkey

**Keywords:** apoptosis, cisplatin, combination, DNA damage, esomeprazole, mitochondrial membrane potential

## Abstract

Proton pump inhibitors (PPIs), including esomeprazole, impact the acidic tumor microenvironment, potentially influencing cancer cell behavior. By examining the combined effects of esomeprazole and cisplatin on SNU‐1 gastric carcinoma cells, this study sought to elucidate the mechanisms through which esomeprazole enhances cisplatin's cytotoxicity, potentially allowing for effective treatment with reduced cisplatin dosages. SNU‐1 cells were treated with varying doses of esomeprazole and cisplatin, alone and in combination. Cell viability was assessed using the XTT assay. Oxidative stress (TAS/TOS), apoptosis (Annexin V, cleaved PARP), mitochondrial membrane potential, and DNA damage (8‐oxo‐dG, γH2AX, ATM) were evaluated using flow cytometry and ELISA. Statistical significance was determined by ANOVA. Esomeprazole alone showed no significant effect on SNU‐1 cell viability, oxidative stress (TAS/TOS), apoptosis, mitochondrial membrane potential, or DNA damage. Cisplatin, however, significantly reduced cell viability (IC50 = 3.024 µg/mL), increased oxidative stress (decreased TAS, increased TOS), diminished apoptosis (increased Annexin V binding and cleaved PARP levels), disrupted mitochondrial membrane potential, and caused significant DNA damage (increased H2AX and ATM phosphorylation, and elevated 8‐oxo‐dG) (*p* < 0.001). Notably, the combination of esomeprazole and cisplatin synergistically enhanced cisplatin's effects. The combination resulted in a significantly greater reduction in cell viability (CI < 1), a further increase in oxidative stress, a higher level of apoptosis, amplified mitochondrial depolarization, and potentiated DNA damage compared to cisplatin alone (*p* < 0.001). Esomeprazole potentiates cisplatin‐induced cytotoxicity in SNU‐1 gastric cancer cells by enhancing oxidative stress, apoptosis, mitochondrial dysfunction, and DNA damage. This suggests a potential therapeutic strategy to improve cisplatin efficacy and overcome resistance in gastric cancer.

## Graphical abstract

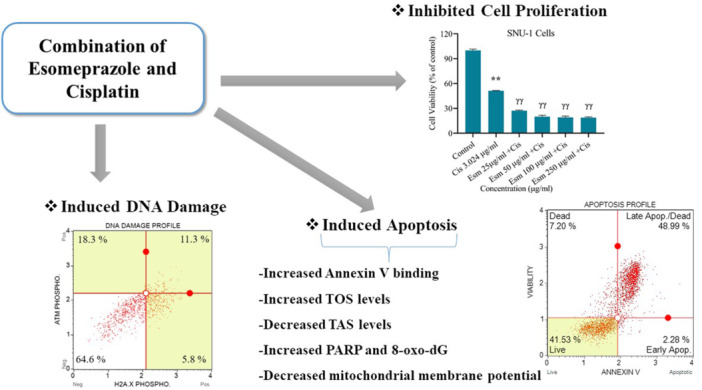

## 1 Introduction

Even with improvements in both identifying and treating it, stomach malignancy persists as a major contributor to global cancer fatalities. Data provided by the World Health Organization (WHO) indicates that this form of cancer ranks among the most frequently diagnosed worldwide and is a significant driver of cancer‐related deaths [1]. The WHO acknowledges *Helicobacter pylori*'s capacity to induce cancer. Research consistently demonstrates that individuals with *H. pylori* infections face roughly double the likelihood of developing gastric adenocarcinoma [2]. Data indicates that the occurrence of stomach cancer is on the decline when *H. pylori* is successfully treated [3].

Proton pump inhibitors (PPIs) diminish stomach acid production by permanently attaching to the H^+^/K^+^‐ATPase pump located on parietal cells, effectively halting its function. PPIs are also used in *H. pylori* eradication therapy. Esomeprazole is the S‐isomer of omeprazole and exhibits greater potency at lower pH levels than pantoprazole and lansoprazole [4]. Cancer cells have a high need for glucose due to their rapid growth. They obtain most of this glucose through glycolysis, a process that produces lactic acid and contributes to the lowered pH of tumors [5]. This acidic microenvironment promotes tumor growth, spread, and resistance to treatment. Additionally, t stimulates angiogenesis, which further fuels tumor development. The survival of tumor cells in an acidic pH is a popular topic in developing new drugs [5]. Using PPIs inhibits H^+^/K^+^‐ATPase function in stomach parietal cells, resulting in a decrease in gastric acid production and a subsequent increase in gastric pH. While some studies have shown that PPI use reduces the risk of gastric cancer, others have indicated an increased risk. In vitro studies by Wenjie et al. have demonstrated that acidic pH enhances the viability, invasion, and metastasis of cancer cells [5]. Proton pumps, which contribute to the regulation of cellular pH and ion balance, are found in many tissues beyond the stomach. One type of proton pump, V‐ATPase, is crucial for tumor cell viability under acidic stress. By expelling hydrogen ions from the cell, V‐ATPase contributes to the acidic environment surrounding the tumor and helps maintain a more alkaline internal environment. Tumor cells also utilize proton pumps to prevent the activation of enzymes that could lead to self‐destruction (autolysis) [6]. The V‐ATPase‐generated hydrogen ion gradient contributes to increased tumor cell invasiveness and metastatic potential, both hallmarks of cancer. The increase in the acidic environment in the tumor microenvironment also results in the formation of chemotherapy resistance [7]. It has been indicated that V‐ATPase inhibition increases cell death in trastuzumab‐resistant breast cancer cells and stops their growth by inhibiting angiogenesis [8]. Acidic extracellular pH and alkaline intracellular pH are common features of most solid tumors. This characteristic of tumor cells is implicated in cisplatin resistance. One of the possible mechanisms of cisplatin‐resistant cancer cells is the overexpression of V‐ATPase [9]. Several studies have shown that PPIs inhibit V‐ATPase and H‐ATPase activity [10]. Modulating the tumor cell microenvironment's pH with certain drugs has enabled new cancer treatment strategies when combined with existing agents. A recent investigation has revealed that administering esomeprazole with paclitaxel in A549/Taxol cells reduces paclitaxel resistance through the augmentation of apoptosis and suppression of cell expression [11]. A 2020 publication revealed that esomeprazole effectively reduced the growth of paclitaxel‐resistant cells, with the degree of inhibition correlating with the dosage [12]. Both studies have shown that esomeprazole exerts its effects by reducing V‐ATPase activity [11, 12].

While PPIs have shown positive effects on cancer cells, the specific impact of combining esomeprazole with cisplatin at varying doses remains unclear. Our study hypothesizes that combining esomeprazole with cisplatin in gastric carcinoma cells will enhance cytotoxicity and reduce cisplatin resistance, allowing for effective treatment with lower cisplatin doses to minimize side effects and resistance development. This study aimed to investigate how esomeprazole influences the cytotoxic effects of cisplatin in SNU‐1 gastric carcinoma cells, with a focus on elucidating the involved mechanisms.

## 2 Chemicals and Consumables

The Gastric Carcinoma (SNU‐1) cell line, penicillin/streptomycin (10,000 U/mL), DMEM/Nutrient Mixture F‐12 Ham (1:1 mixture), Fetal Bovine Serum (FBS), Trypsin‐EDTA solution, esomeprazole, cisplatin, and various consumables necessary for cell culture were used.

### 2.1 Cell Culture

Gastric carcinoma cells (SNU‐1), obtained from ATCC, were cultivated aseptically at 37°C and 5% CO_2_ in 25 cm^2^ flasks, in DMEM: F12 (1:1) cell culture medium containing 1% l‐glutamine, 1% penicillin‐streptomycin, and 10% fetal bovine serum. Upon achieving 80% confluency, cells were passaged. Experiments commenced after the third passage. Esomeprazole was selected for this study on the SNU‐1 gastric carcinoma cell line due to several key factors: the widespread clinical use of PPIs, their established safety profile with minimal side effects even at elevated doses, their cost‐effectiveness, and documented efficacy in conditions such as Zollinger‐Ellison syndrome, where high‐dose PPI therapy is routinely employed. The doses used for esomeprazole were determined as 25, 50, 100, 200, and 250 µg/mL [11, 12, 13]. The doses used for cisplatin were chosen as 1, 2.5, 5, 10, 25, 50, 100, and 200 µg/mL [14].

### 2.2 Cell Viability Measurement Using XTT

The effect of combined esomeprazole and cisplatin treatment on the viability of human gastric carcinoma cells was investigated using the XTT (2,3‐bis(2‐methoxy‐4‐nitro‐5‐sulfophenyl)‐5‐[(phenylamino)carbonyl]‐2H‐tetrazolium hydroxide) assay. The assay relies on the ability of living cells to convert XTT, a tetrazolium salt, into a colored formazan product through metabolic reduction. The resulting dye is water‐soluble, and its intensity can be measured spectrophotometrically at specific wavelengths. The intensity of the orange color is directly related to the quantity of metabolically active cells. SNU‐1 cells were plated at 10,000 cells per well in 96‐well plates. At their growth phase, cells were treated with cisplatin and esomeprazole, both alone and in combination, at various doses for 48 h. After establishing the cisplatin IC50, increasing esomeprazole concentrations were applied, followed by a fixed cisplatin concentration (IC50) 2 h later. After incubation, 50 µL XTT solution was added to each well, and the plates were incubated for 4 h in CO_2_. Using a microplate reader, the absorbance of the solution in each well was measured at a wavelength of 450 nm. The cell viability ratio was calculated using the formula: %Cell Viability = (Drug O.D./Control O.D.) × 100, considering the cell viability of the control group as 100% [15, 16]. To assess the combined effects of multiple compounds, the combination index (CI) was calculated using the IC50 values of individual drugs and the drug combination, following the method described by Chou and Talalay [17, 18].

### 2.3 Measurement of Total Antioxidant Status (TAS) and Total Oxidant Status (TOS)

TAS and TOS levels will be measured using commercially available kits to evaluate the effects of post‐treatment esomeprazole on oxidative and nitrosative stress in cells. The procedure will follow the manufacturer's instructions. We explored this method in our previous studies [19, 20].

### 2.4 Annexin V Staining

SNU‐1 cells (1 × 10⁶ cells/well) were grown in six‐well plates for 48 h before being treated with cisplatin (3.024 μg/mL), esomeprazole (25 μg/mL), or their combination. Following incubation, cells were gathered, rinsed with PBS, and analyzed for apoptosis using the Muse Cell Analyzer (Millipore) with the Annexin V assay kit (Millipore), according to the manufacturer's instructions. The percentage of apoptotic cells was then determined. This method was explored in previous studies [21, 22].

### 2.5 Assessing Mitochondrial Membrane Potential Using Flow Cytometry

SNU‐1 cells (5 × 10^6^ cells/well) were seeded in six‐well plates and allowed to adhere overnight before assessing mitochondrial transmembrane potential with the Muse MitoPotential Kit. After 48 h of incubation with cisplatin (3.024 µg/mL), esomeprazole (25 µg/mL), or the combined treatment, cells were collected and resuspended in 100 µL of media. Mitochondrial membrane potential was then assessed by staining with Muse MitoPotential Dye, followed by a 20‐min incubation at 37°C. Subsequently, 5 µL of Muse MitoPotential 7‐AAD reagent was added, and the samples were incubated for 5 min at room temperature, shielded from light. The proportions of live, depolarized, depolarized/dead, and dead cells were then determined using the Muse Cell Analyzer (Merck Millipore) [15].

### 2.6 DNA Damage Assay

To determine the activation of ATM and H2AX, a measure of DNA damage, SNU‐1 cells were subjected to various treatments: cisplatin (3.024 µg/mL), esomeprazole (25 µg/mL), or a combined application of both. Following a 48‐h incubation at 37°C, cells were prepared for analysis using the Muse Multi‐Color DNA Damage Kit (Merck Millipore), strictly adhering to the manufacturer's protocol. Initially, cells underwent a centrifugation and PBS wash, followed by resuspension in assay buffer. Subsequently, a fixation step was performed, involving incubation on ice for 10 min. After another wash, cells were permeabilized using an ice‐cold buffer. An antibody cocktail (10 µL) was then introduced to each cell suspension, with a 30‐min dark incubation at room temperature. A final centrifugation and wash preceded resuspension in 200 µL of assay buffer. Finally, the proportions of cells exhibiting no DNA damage, ATM activation, H2AX activation, and double‐strand DNA breaks were quantified using the Muse Multi‐Color DNA Damage software module (Millipore) [23, 24].

### 2.7 Determination of Cleaved PARP, and 8‐Hydroxy‐Deoxyguanosine Levels

To quantify cleaved PARP and 8‐oxo‐dG, SNU‐1 cells (10⁶ cells/well) were placed in six‐well plates and treated for 48 h with cisplatin (3.024 µg/mL), esomeprazole (25 µg/mL), or their combination. Subsequently, specific human ELISA kits (BT Lab Shanghai, China) were utilized for analysis. Following treatment, cells were collected, resuspended in PBS, and cellular damage was induced via multiple freeze‐thaw cycles. Following lysate preparation, marker levels were quantified per the manufacturer's protocol. Total protein was assessed using the Pierce BCA assay [25].

### 2.8 Statistical Processing

Data are expressed as mean ± SEM and analyzed by one‐way ANOVA with Tukey's post hoc test. Statistical significance was set at *p* < 0.05. SPSS v.22 was used for all analyses.

## 3 Results

### 3.1 The Effect of Esomeprazole, Cisplatin, and Their Combination on SNU‐1 Cell Viability

**Figure 1:**
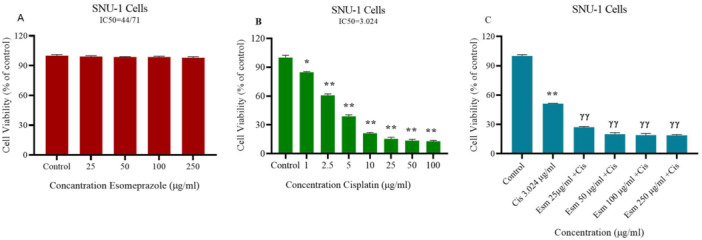
SNU‐1 cell viability after treatment with esomeprazole, cisplatin, and combination. Cells were treated for 48 h with (A) esomeprazole (25–250 µg/mL), (B) cisplatin (1–100 µg/mL), and (C) esomeprazole (25–250 µg/mL) + cisplatin (3.024 µg/mL, IC50). Viability was determined by XTT assay (percentage of live cells vs. control; mean ± SEM, *n* = 3). *p < 0.01, **p < 0.001 versus control; ^ɣɣ^ *p* < 0.001 versus cisplatin.

**Figure 2:**
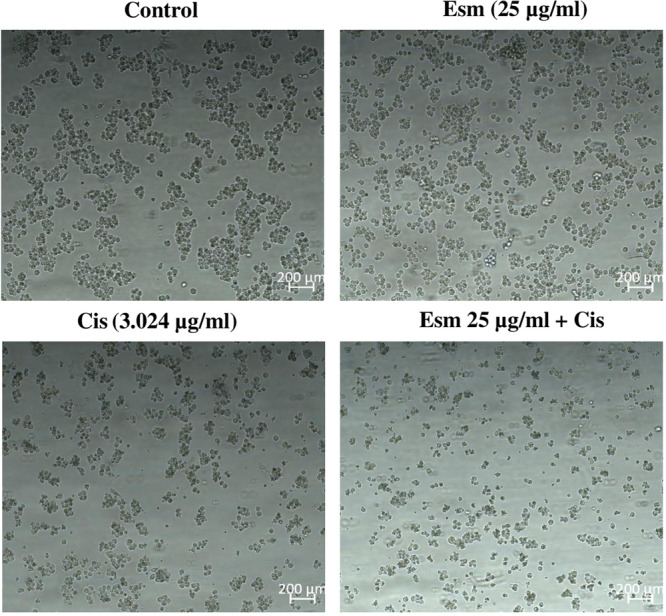
Light microscopic images of cells in the control, esomeprazole, cisplatin, and esomeprazole + cisplatin groups. The incubation time was 48.

Cisplatin (1−200 µg/mL), esomeprazole (500−25 µg/mL), and their combination (esomeprazole at 500−25 µg/mL + cisplatin at its IC50 of 3.024 µg/mL) were evaluated for their effects on SNU‐1 cell viability using the XTT assay. Treatment with varying doses of esomeprazole did not affect cell viability and showed no significant variation from the control (Figure 1A). In contrast, treatment with varying doses of cisplatin, starting from the lowest dose, exhibited cytotoxicity and showed a statistically significant variation from the control (*p* < 0.001) (Figure 1B). The IC50 dose of cisplatin was calculated to be 3.024 µg/mL. Finally, treatment with varying doses of esomeprazole in combination with a fixed dose of cisplatin demonstrated dose‐independent cytotoxicity and revealed a statistically significant variation from the control (*p* < 0.001) (Figure 1C). The combination effect was evaluated by calculating the CI. All esomeprazole and cisplatin combinations yielded a CI value of less than 1, indicating a synergistic effect. Figure 2 illustrates the light microscopic differences observed in the esomeprazole, cisplatin, and combination treatment groups compared to the control.

### 3.2 Impact of Esomeprazole, Cisplatin, and Combined Treatment on TAS and TOS

**Figure 3:**
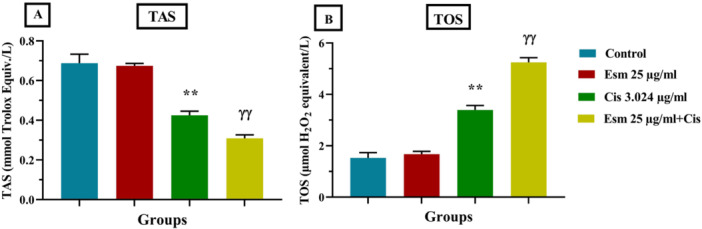
Impact of esomeprazole (25 µg/mL), cisplatin (3.024 µg/mL), and esomeprazole (25 µg/mL) + cisplatin (3.024 µg/mL) on TAS (A) and TOS (B) compared to the control (mean ± SEM, *n* = 3). The incubation time was 48. **p < 0.001, versus control; ^ɣɣ^ *p* < 0.001 versus cisplatin.

The effects of cisplatin (3.024 µg/mL), esomeprazole (25 µg/mL), and their combination (25 µg/mL esomeprazole + 3.024 µg/mL cisplatin) on oxidative stress markers (TAS and TOS) were evaluated in SNU‐1 gastric cancer cells. Esomeprazole treatment alone did not significantly alter TAS or TOS levels compared to the control group (Figure 3). Cisplatin, however, significantly reduced TAS levels and increased TOS levels compared to the control (*p* < 0.001) (Figure 3), indicating increased oxidative stress. Notably, the combination of esomeprazole and cisplatin resulted in a significantly greater reduction in TAS levels and a significantly higher elevation of TOS levels compared to cisplatin alone (*p* < 0.001) (Figure 3), suggesting a synergistic increase in oxidative stress.

### 3.3 The Apoptotic Effect of Esomeprazole, Cisplatin, and Combined Treatment on SNU‐1 Cells

**Figure 4:**
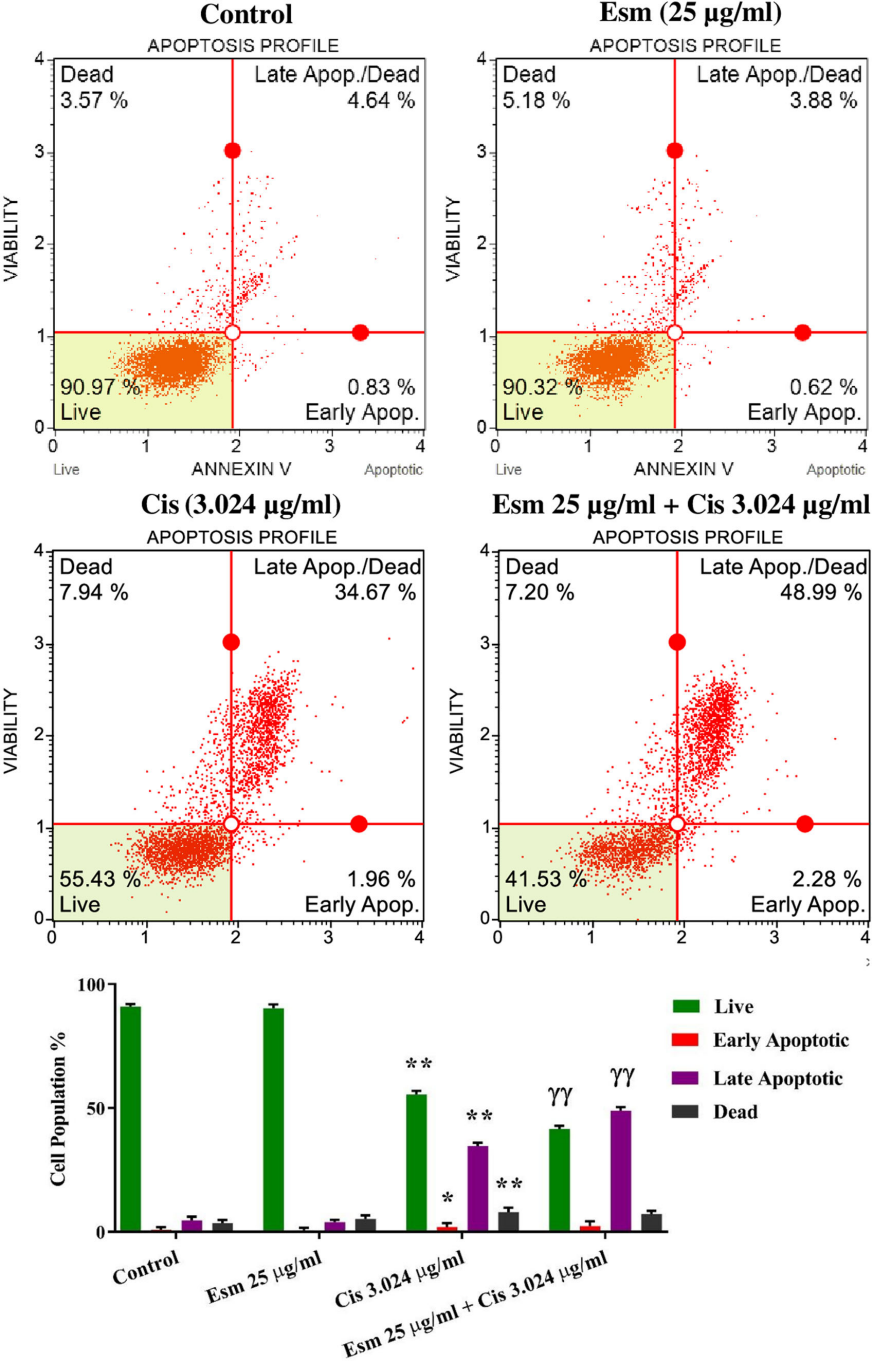
Evaluation of the effects of esomeprazole (25 µg/mL), cisplatin (3.024 µg/mL), and esomeprazole (25 µg/mL) + cisplatin (3.024 µg/mL) compared to the control on the apoptosis profile using flow cytometry (mean ± SEM, *n* = 3). The incubation time was 48. *p < 0.05, **p < 0.001, versus control; ^ɣɣ^ *p* < 0.001 versus cisplatin.

Flow cytometry was utilized to evaluate the apoptosis profile of cisplatin (3.024 µg/mL), esomeprazole (25 µg/mL), and their combination (25 µg/mL esomeprazole + 3.024 µg/mL cisplatin) on SNU‐1 cells. As seen in Figure 4, esomeprazole alone had no discernible impact on the apoptotic rate of SNU‐1 cells when contrasted with the control. Conversely, cisplatin treatment substantially increased SNU‐1 cell apoptosis (*p* < 0.001, Figure 4), demonstrating its antiproliferative action through apoptotic mechanisms. Interestingly, the combined application of esomeprazole and cisplatin produced a considerably higher level of SNU‐1 cell apoptosis than cisplatin used alone (*p* < 0.001, Figure 4), implying that esomeprazole enhances the apoptotic susceptibility of SNU‐1 cells to cisplatin.

### 3.4 The Effect of Esomeprazole, Cisplatin, and Combined Treatment on Mitochondrial Membrane Potential in SNU‐1 Cells

**Figure 5:**
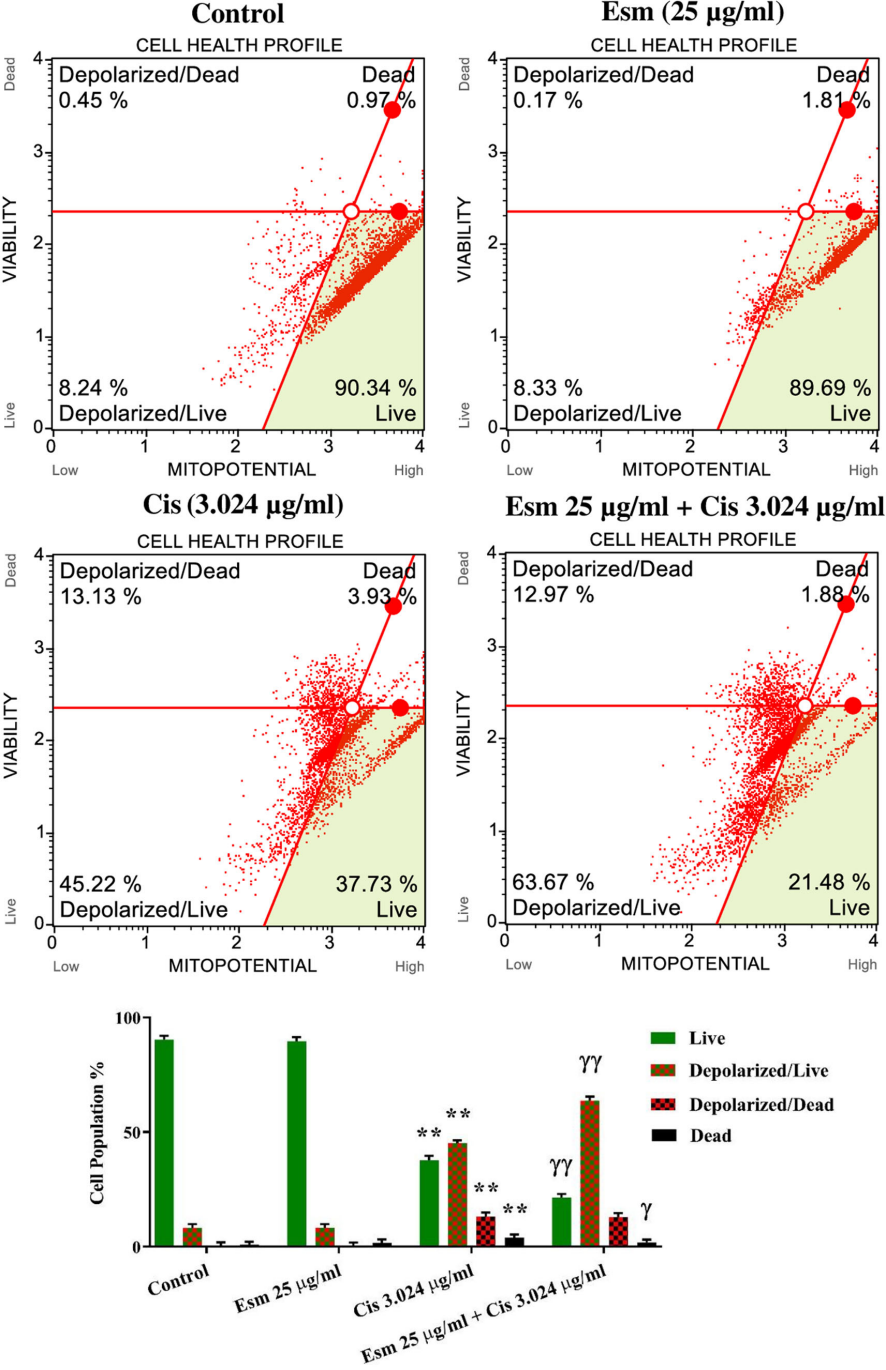
Evaluation of the effects of esomeprazole (25 µg/mL), cisplatin (3.024 µg/mL), and esomeprazole (25 µg/mL) + cisplatin (3.024 µg/mL) compared to the control on mitochondrial membrane potential using flow cytometry (mean ± SEM, *n* = 3). The incubation time was 48. **p < 0.001, versus control; ^ɣ^ *p* < 0.05, ^ɣɣ^ *p* < 0.001 versus cisplatin.

Flow cytometry was utilized to analyze the changes in mitochondrial membrane potential within SNU‐1 gastric cancer cells following treatment with cisplatin (3.024 µg/mL), esomeprazole (25 µg/mL), or their combined administration. While esomeprazole alone showed no discernible impact on the proportion of cells with depolarized mitochondria relative to viable cells, cisplatin induced a substantial shift towards mitochondrial depolarization, a hallmark of apoptosis (*p* < 0.001, Figure 5). Combining esomeprazole with cisplatin amplified this depolarization effect significantly beyond that observed with cisplatin alone (*p* < 0.001, Figure 5). This suggests that esomeprazole may enhance cisplatin's ability to disrupt mitochondrial function and promote cell death in SNU‐1 cells.

### 3.5 Effect of DNA Damage Response of Esomeprazole, Cisplatin, and Combined Treatment on SNU‐1 Cells

**Figure 6:**
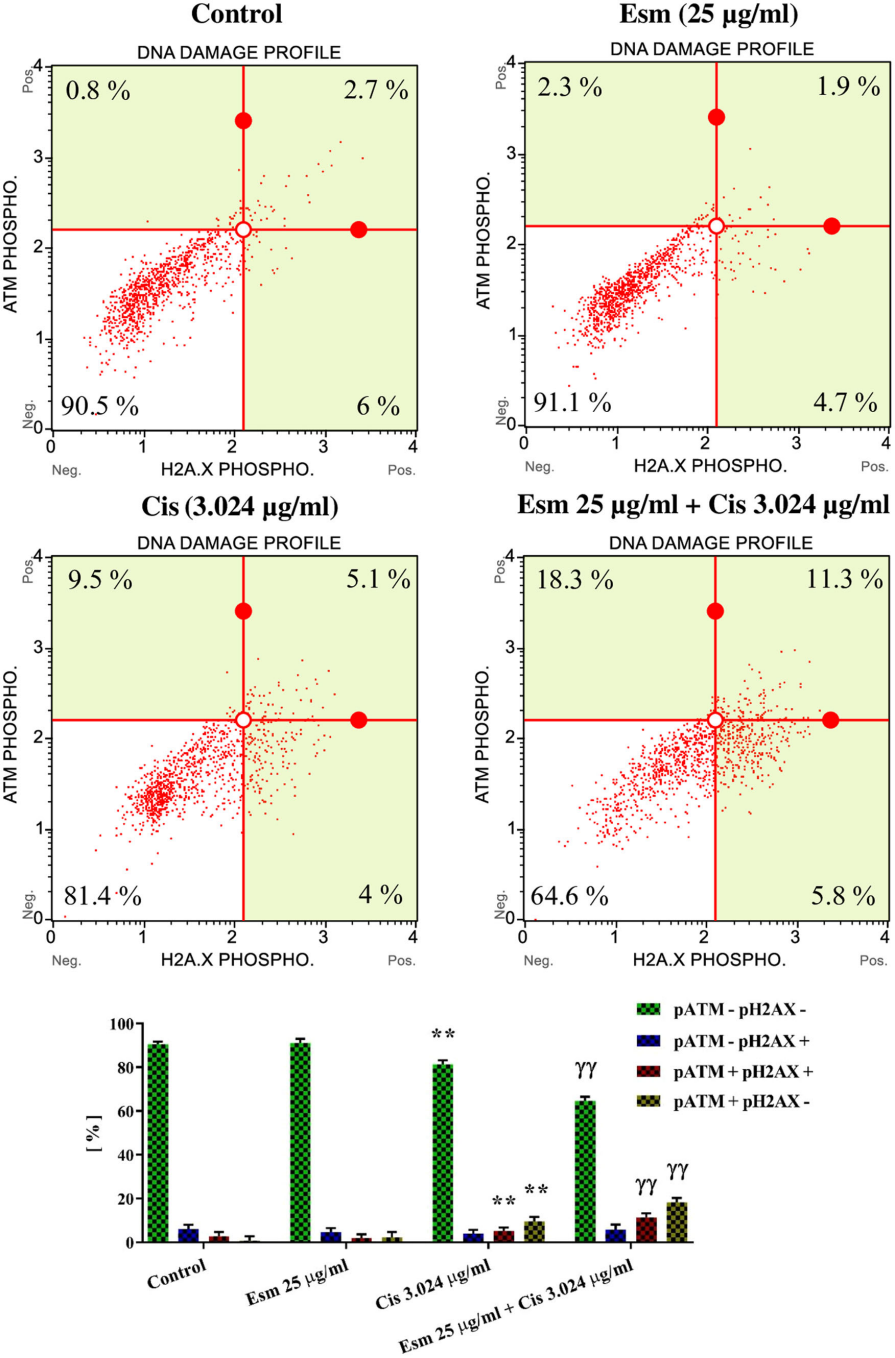
Evaluation of the effects of esomeprazole (25 µg/mL), cisplatin (3.024 µg/mL), and esomeprazole (25 µg/mL) + cisplatin (3.024 µg/mL) compared to the control on DNA damage, and activation of ATM and H2AX using flow cytometry (mean ± SEM, *n* = 3). The incubation time was 48. **p < 0.001, versus control; ^ɣɣ^ *p* < 0.001 versus cisplatin.

SNU‐1 cells were treated with cisplatin (3.024 µg/mL), esomeprazole (25 µg/mL), or their combined administration for 48 h, and a DNA damage assay was performed to investigate the potential DNA‐damaging effects of these treatments. As evidenced in Figure 6, esomeprazole alone did not elicit measurable DNA damage. Conversely, cisplatin treatment significantly elevated H2AX and ATM phosphorylation levels (*p* < 0.001, Figure 6), signifying its DNA‐damaging potential. Notably, the combined administration of esomeprazole and cisplatin further augmented the phosphorylation of both H2AX and ATM compared to cisplatin alone (*p* < 0.001, Figure 6), suggesting that esomeprazole potentiates cisplatin‐induced DNA lesions.

### 3.6 Impact of Esomeprazole, Cisplatin, and Combined Treatment on Cleaved PARP, and 8‐Hydroxy‐Deoxyguanosine (8‐oxo‐dG)

**Figure 7:**
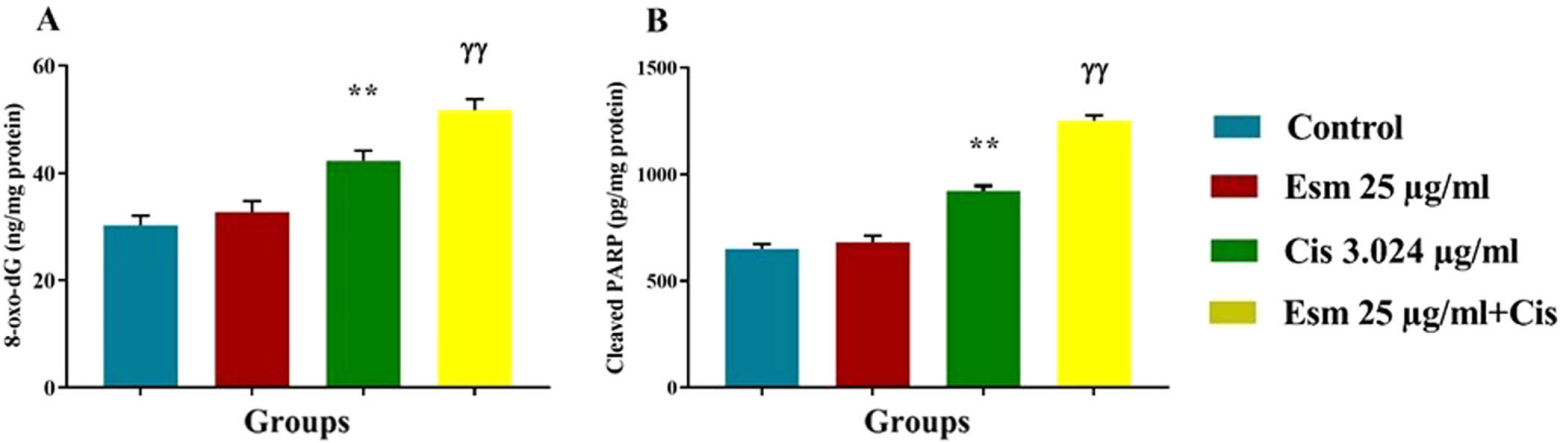
Impact of esomeprazole (25 µg/mL), cisplatin (3.024 µg/mL), and esomeprazole (25 µg/mL) + cisplatin (3.024 µg/mL) on cleaved PARP (A) and 8‐oxo‐dG (B) compared to the control (mean ± SEM, *n* = 6). The incubation time was 48. **p < 0.001, versus control; ^ɣɣ^ *p* < 0.001 versus cisplatin.

ELISA kits were used to evaluate the effects of cisplatin (3.024 µg/mL), esomeprazole (25 µg/mL), and their combination on the apoptotic marker cleaved PARP and the DNA damage marker 8‐oxo‐dG in SNU‐1 gastric cancer cells. According to Figure 7, esomeprazole did not significantly alter cleaved PARP and 8‐oxo‐dG levels when compared to the control group. Cisplatin significantly increased both markers (*p* < 0.001, Figure 7), demonstrating its DNA‐damaging and apoptotic effects. Notably, a significantly higher elevation of cleaved PARP and 8‐oxo‐dG levels was observed with the combination of esomeprazole and cisplatin compared to cisplatin alone (*p* < 0.001, Figure 7), indicating esomeprazole's potentiation of cisplatin‐induced apoptosis and DNA damage.

## 4 Discussion

Drug resistance remains the primary obstacle to effective chemotherapy in gastric cancer, despite advances in treatment and diagnosis [26]. Recent studies highlight the role of the V‐ATPase‐mediated acidic tumor microenvironment in cancer drug resistance [27]. Nausea and vomiting caused by chemotherapy pose a significant challenge to effective cancer treatment [28]. PPIs, effective V‐ATPase inhibitors, are widely used for chemotherapy‐related nausea and vomiting [29]. Therefore, investigating the impact of PPIs on chemotherapy‐based gastric cancer treatment is crucial. This study is the first to demonstrate that esomeprazole enhances the cytotoxic effect of cisplatin on SNU‐1 gastric cancer cells. While esomeprazole alone did not exhibit antitumor activity in these cells, it increased their sensitivity to cisplatin. These findings suggest that esomeprazole may improve the effectiveness of cisplatin‐based chemotherapy in gastric cancer patients. Consistent with our findings, Matsumura et al. reported that lansoprazole and esomeprazole significantly enhanced the cytotoxic effect of 5‐FU in esophageal cancer cell lines. Clinically, patients treated with PPIs demonstrated better overall survival compared to those who did not receive PPI treatment [30]. Luciani et al. also demonstrated that pretreatment with omeprazole, esomeprazole, or pantoprazole sensitized human melanoma, adenocarcinoma, and lymphoma cell lines to cisplatin, 5‐fluorouracil, and vinblastine, reducing IC50 values by up to two orders of magnitude. This PPI pretreatment was associated with V‐H^+^‐ATPase inhibition and increased extracellular and lysosomal pH [31]. Many anticancer drugs induce oxidative stress, damaging cellular components like proteins, lipids, and DNA. Oxidative stress is often measured using TOS [32], while TAS measures the body's ability to combat this damage [15]. By measuring TAS and TOS levels, this study investigated whether combining esomeprazole and cisplatin alters oxidative stress levels. Results showed that a 48‐h exposure to the combination significantly increased TOS levels while simultaneously decreasing TAS levels compared to cells treated with cisplatin alone. These findings suggest that esomeprazole enhances cisplatin‐induced oxidative stress in SNU‐1 cells, evidenced by the greater increase in TOS and decrease in TAS compared to cisplatin‐only treatment. Apoptosis, or programmed cell death, is crucial for controlling cell growth. Triggered by specific signals, it causes distinct changes: phosphatidylserine (PS) externalization, protein cleavage, chromatin condensation and fragmentation, and eventually, membrane degradation [33, 34]. Cancer develops when apoptosis malfunctions, contributing to tumor growth and therapy resistance. A promising cancer treatment strategy involves manipulating apoptosis to kill cancer cells. Many anticancer drugs work by inducing apoptosis, selectively eliminating cancer cells while sparing healthy ones, thus shrinking tumors and preventing metastasis [25, 35]. Annexin V, a protein that binds to PS, is a common marker for apoptosis. In early apoptosis, PS appears on the cell's outer surface, allowing Annexin V to bind and thus indicate cell death easily [36]. This study used flow cytometry to investigate whether esomeprazole enhances cisplatin‐induced apoptosis in SNU‐1 cells. ELISA was also used to confirm the combination's apoptotic effect on SNU‐1 cells by measuring cleaved PARP levels. PARP, a key protein in DNA base excision repair, is a known caspase substrate; its cleavage or inhibition can induce cell death by exploiting DNA repair deficiencies [37]. Our findings suggest that esomeprazole enhanced cisplatin‐induced apoptosis, evidenced by increased Annexin V binding and cleaved PARP levels compared to cisplatin treatment alone. To determine if the combination treatment's cytotoxic effects were mediated by DNA damage, we employed flow cytometry and 8‐oxo‐dG ELISA to quantify DNA damage in SNU‐1 cells. Compared to cisplatin alone, the combination treatment significantly increased DNA damage in SNU‐1 cells, as shown by elevated 8‐oxo‐dG, γH2AX, and ATM phosphorylation. 8‐oxo‐dG, a marker of oxidative stress, indicates increased DNA lesions from reactive oxygen species (ROS), contributing to instability [38]. Concurrently, increased γH2AX and ATM phosphorylation signify a robust double‐strand break response, where ATM activates H2AX to recruit repair proteins and trigger cell cycle checkpoints [39]. This combined increase in oxidative damage and double‐strand breaks suggests the combination treatment severely compromised genomic integrity, likely overwhelming repair mechanisms and leading to DNA fragmentation and apoptosis. Consequently, the enhanced cytotoxic and apoptotic effects of the combination therapy are attributable to its potent induction of DNA damage, demonstrated by increased 8‐oxo‐dG and activation of the ATM‐H2AX pathway.

Mitochondria, the cell's powerhouses, are key to energy balance and produce ROS. They also regulate apoptosis. Mitochondrial changes reflect cellular health and stress. Healthy mitochondria maintain a transmembrane potential that drives ATP synthesis. This potential is lost in early apoptosis, often due to mitochondrial permeability transition pore opening and cytochrome c release, triggering apoptosis. Changes in this potential are linked to various cell death processes. Loss of mitochondrial membrane potential indicates dysfunction and is crucial in apoptosis, toxicity, and disease studies. Excessive ROS and mitochondrial damage activate the intrinsic apoptosis pathway [40]. Our findings on mitochondrial membrane potential suggest that esomeprazole exacerbated cisplatin‐induced mitochondrial membrane damage. Consistent with our results, Duan et al. found that esomeprazole and cisplatin together synergistically suppressed the proliferation, invasion, and migration of cisplatin‐resistant ovarian cancer cells. They proposed that this synergy might stem from enhanced suppression of c‐MYC, EMT, and the AKT/mTOR pathway, alongside increased pro‐apoptotic BAX and cleaved PARP levels. Furthermore, their research indicated that the combination also synergistically increased the expression of the DNA damage marker γH2A.X [41]. Chi et al. demonstrated that combining esomeprazole with paclitaxel significantly lowered the IC50, indicating a more potent inhibitory effect compared to individual treatments. This combination also dramatically increased apoptosis levels. Furthermore, the esomeprazole and paclitaxel group demonstrated a marked decrease in Bcl‐2 and P‐gp expression, while bcl‐xl protein expression increased. Finally, this combined treatment led to a significant reduction in intracellular pH [12]. Wang et al. found that PPIs can hinder DNA damage repair in breast cancer, making cancer cells more susceptible to treatment. This effect is achieved by the inhibitors targeting fatty acid synthase [42]. This study demonstrates that combining esomeprazole with cisplatin significantly enhances SNU‐1 gastric cancer cell growth suppression. The synergistic effect appears to stem from a multifaceted mechanism: the combination therapy intensifies oxidative stress, evidenced by elevated TOS and diminished TAS, thus disrupting the cellular redox balance. Consequently, apoptosis is potently induced, marked by increased annexin V binding and heightened cleaved PARP levels—all hallmarks of programmed cell death. Furthermore, the combination triggers substantial DNA damage, as indicated by increased 8‐oxo‐dG levels, and compromises mitochondrial membrane integrity, a critical factor in initiating apoptosis. In essence, this study illuminates a promising therapeutic strategy to bolster the effectiveness of cisplatin, potentially offering a way to combat resistance and improve treatment outcomes in gastric cancer. Further research is warranted to explore this combination's precise molecular pathways and clinical applicability. While our findings suggest that esomeprazole enhances cisplatin's anticancer effect in SNU‐1 cells, potentially through increased oxidative stress, apoptosis, mitochondrial dysfunction, and DNA damage, a limitation of this study is the lack of direct measurements of V‐H⁺‐ATPase expression and activity. Therefore, the proposed mechanism involving V‐H⁺‐ATPase remains to be fully elucidated in this cellular context. Future studies should aim to investigate the expression and activity levels of V‐H⁺‐ATPase to directly assess its contribution to the observed effects. Another limitation of our study is that we did not evaluate the cytotoxic effects of the esomeprazole and cisplatin combination on normal cells. While our findings indicate that esomeprazole alone had minimal impact on cancer cell viability, and the observed synergy with cisplatin could potentially allow for lower doses of cisplatin, the safety profile of this combination on normal cells remains to be fully characterized. To better assess the treatment's selectivity and safety, future investigations should include evaluating the cytotoxicity of this combination in relevant normal cell lines.

## Author Contributions

Z.J. and F.Y. contributed to the study's conceptualization, methodology, investigation, resource provision, and writing (original draft, review, and editing). M.G., M.E., and O.K. were responsible for conceptualization, formal analysis, validation, visualization, and methodology.

## Conflicts of Interest

The authors declare no conflicts of interest.

## Data Availability

The data that support the findings of this study are available from the corresponding author upon reasonable request.
